# *Maesa ramentacea* Leaf Extract Protects Against Gentamicin‐Induced Nephrotoxicity in Wistar Rats by Modulating Renal Oxidative Stress, Inflammation and Apoptosis

**DOI:** 10.1155/vmi/6127118

**Published:** 2026-05-14

**Authors:** Fahmida Akter, Md Shafiqul Islam Sovon, Baizid Hossain, Md Ariful Islam, Subrato Das, Anik Biswas, Md. Sohorab Uddin, Md. Mahmudul Hasan

**Affiliations:** ^1^ University of Chittagong, Chittagong, 4331, Bangladesh, cu.ac.bd; ^2^ University of Dhaka, Dhaka, 1000, Bangladesh, du.ac.bd; ^3^ Jahangirnagar University, Dhaka, 1342, Bangladesh, juniv.edu; ^4^ University of Central Florida, Orlando, 32816, Florida, USA, ucf.edu; ^5^ University of Southern Mississippi, Hattiesburg, 39406, Mississippi, USA, usm.edu

**Keywords:** acute kidney injury, antioxidant enzymes, apoptosis, inflammation, *Maesa ramentacea*, oxidative stress

## Abstract

**Background:**

Gentamicin‐induced nephrotoxicity continues to be a significant clinical concern, while effective and safe protective options remain limited. The nephroprotective potential of *Maesa ramentacea* has not yet been explored. To address this gap, the present study investigated the ethanolic leaf extract of *M. ramentacea* (MR‐LEE) for its effects on gentamicin‐induced kidney injury in Wistar rats.

**Methods:**

GC‐MS profiling was employed to identify bioactive metabolites. Rats were categorised into five groups: control, gentamicin (100 mg/kg), gentamicin combined with silymarin (100 mg/kg), gentamicin combined with MR‐LEE (200 mg/kg) and gentamicin combined with MR‐LEE (400 mg/kg). Renal biomarkers, oxidative stress indicators, inflammatory and apoptotic markers and histopathological evaluations were conducted.

**Results:**

Nine bioactive metabolites have been identified using GC‐MS. Gentamicin significantly elevated serum creatinine (1.17 ± 0.13 mg/dL), urea (69.9 ± 3.44 mg/dL), uric acid (5.52 ± 1.15 mg/dL) and LDH (4770 ± 480 U/L) compared to the control group (*p* = 0.0001). MR‐LEE at 400 mg/kg significantly decreased creatinine (0.65 ± 0.05 mg/dL), urea (45.3 ± 2.66 mg/dL) and LDH (4250 ± 380 U/L) (*p* = 0.0001), approaching the levels observed with silymarin. Gentamicin increased TNF‐α (58.8 ± 4.6 pg/mg), IL‐6 (53.1 ± 2.23 pg/mg) and caspase‐3 (37.6 ± 2.44 ng/mg) (*p* = 0.0001), but MR‐LEE 400 mg/kg significantly decreased these levels to 33.1 ± 2.3, 29.5 ± 2.33 and 21.5 ± 1.66, respectively (*p* = 0.005). MR‐LEE restored antioxidant enzyme levels (GSH, SOD and CAT) and reduced lipid peroxidation, as evidenced by histological analyses showing decreased tubular necrosis and nearly normal renal architecture.

**Conclusion:**

MR‐LEE appeared to exert a notable, dose‐dependent nephroprotective effect. Future research should focus on isolating active components and elucidating molecular mechanisms to facilitate translational use.

## 1. Introduction

Nephrotoxicity refers to kidney damage induced by pharmaceuticals or toxins, frequently leading to both structural and functional impairment of the kidneys [1]. It generally encompasses tubular necrosis, glomerular damage and modified renal biomarkers, including creatinine and urea [2].

Drug‐induced acute kidney injury (DI‐AKI) remains a significant clinical challenge worldwide. Recent large cohort data indicate that DI‐AKI accounts for nearly 20% of all AKI cases among hospitalised patients, making it a leading contributor to worsening renal outcomes and increased healthcare burden [3]. Specific to aminoglycoside antibiotics, a retrospective study found that 6.1% of hospitalised patients exposed to these agents developed AKI, with intensive care admission, diabetes and concomitant medication use identified as key risk factors [4]. Moreover, observational data suggest that around 20% of AKI cases in clinical practice are directly related to nephrotoxic exposures, including antibiotics, nonsteroidal anti‐inflammatory drugs and contrast agents [5]. The condition is especially common among hospitalised, elderly and critically ill patients, resulting in heightened morbidity, mortality and healthcare expenses [3].

Gentamicin (GEN) accumulates in proximal tubular epithelial cells via endocytic receptors, impairing mitochondrial and endoplasmic reticulum function. This results in excessive production of reactive oxygen species, depletion of antioxidant defences, lipid peroxidation and structural damage to tubular cells [3, 6]. The resultant oxidative stress incites inflammatory responses, elevates cytokine levels and activates apoptotic and necrotic pathways, ultimately leading to tubular necrosis and compromised renal function [7, 8].

Synthetic nephroprotective agents have been investigated to alleviate GEN‐induced renal injury; however, their effectiveness is often variable, and safety concerns limit clinical application [9]. Numerous compounds target a single pathway, whereas nephrotoxicity encompasses multiple interrelated mechanisms [10]. Conversely, natural products provide multitargeted effects. Compounds derived from plants, which are abundant in flavonoids, polyphenols and tannins, can neutralise free radicals, restore antioxidant activity and mitigate oxidative stress and inflammation [11]. Numerous medicinal plants have shown enhancement in renal function and histological preservation in experimental models, underscoring their potential as safer alternatives [12].

Plant‐derived compounds show nephroprotective effects by reducing oxidative stress and inflammation. They also regulate pathways involved in renal fibrosis and apoptosis. Bioactive compounds such as flavonoids, phenolic acids and triterpenoid saponins help protect kidney function and maintain cellular integrity under nephrotoxic conditions [11]. In this context, *Maesa ramentacea* has gained attention for its ethnomedicinal and pharmacological importance. It is traditionally used in Southeast Asia, where its leaf extract is applied to treat diarrhoea in children [13, 14]. Moreover, species of the *Maesa* genus are known to contain abundant triterpenoid saponins and phenolic compounds, which exhibit antibacterial, antifungal, antioxidant, anti‐inflammatory and cytoprotective activities [15, 16]. Given that these phytochemical classes are frequently implicated in nephroprotection, *M. ramentacea* may possess similar renal protective potential. However, despite this phytochemical promise, no direct studies have yet established its nephroprotective efficacy, and its in vivo effects and underlying mechanisms in renal injury remain to be elucidated.

Notwithstanding these encouraging attributes, the nephroprotective potential of *M. ramentacea* remains largely unexplored. Notably, similar mechanistic axes have been reported in other acute kidney injury models, including renal ischemia/reperfusion injury, where oxidative stress, inflammatory cytokines and apoptosis play central roles in tissue damage. This broader mechanistic overlap strengthens the rationale for investigating multitarget natural products in GEN‐induced nephrotoxicity. To address this gap, the present study systematically evaluates the protective effects of the ethanolic leaf extract of *M. ramentacea* (MR‐LEE) against GEN‐induced renal damage in Wistar rats, providing the first in vivo evidence in this context. The study is designed with clearly defined primary outcomes, including biochemical markers of renal function (serum creatinine, urea and uric acid) and histopathological alterations in kidney tissue. Secondary outcomes include oxidative stress parameters (superoxide dismutase [SOD], catalase [CAT], glutathione [GSH] and malondialdehyde [MDA]), inflammatory cytokines (TNF‐α and IL‐6) and the apoptotic marker caspase‐3. By integrating these endpoints, the study comprehensively characterises the nephroprotective effects of MR‐LEE, elucidates its underlying mechanisms and highlights its potential as a natural source of bioactive compounds for mitigating aminoglycoside‐induced nephrotoxicity.

## 2. Materials and Methods

### 2.1. Plant Collection and Extraction

Leaves of *M. ramentacea* were collected from Chittagong, Bangladesh, and authenticated by Professor Sheikh Boktair Uddin, a taxonomist in the Department of Botany at the University of Chittagong, with the voucher specimen deposited under number MMH‐062025(A). The leaves were washed and shade‐dried for 5–7 days and then coarsely ground. They were extracted with 95% ethanol, which efficiently isolates both polar and nonpolar compounds, using a Soxhlet apparatus at 60°C for 24 h. The extract was filtered, concentrated with a rotary evaporator and dried at 50°C. MR‐LEE was stored at 4°C, with a yield of 2.3% [17].

### 2.2. Experimental Animals

Adult Wistar rats, weighing 150–180 g, were obtained from the institutional animal facility. The subjects were maintained under regulated environmental conditions at 22 ± 2°C, with a 12‐hour light/dark cycle, and provided with unrestricted access to standard pellet feed and water. All experimental techniques received approval from the Institutional Animal Ethics Committee (Approval No: AERB‐FBSCU‐20250615‐(1)) and were conducted in accordance with the ARRIVE criteria.

### 2.3. Phytochemical Analysis by Gas Chromatography‐Mass Spectroscopy (GC‐MS)

MR‐LEE was examined with a Shimadzu GC‐17A in conjunction with a TQ 8040 mass spectrometer operating in electron ionisation mode. Helium served as the carrier gas at a flow rate of 0.6 mL/min, with an interface temperature of 280°C and a scan range of 40–350 amu. Compounds were identified utilising the NIST GC‐MS library (Version 08‐S) [18].

### 2.4. Acute Toxicity Study

The acute oral toxicity of MR‐LEE was assessed in male and female Wistar rats in accordance with OECD Guideline 423 [19]. Three groups (*n* = 3 per group) were given MR‐LEE orally at 1000, 2000 and 4000 mg/kg body weight, whereas the control group received distilled water at 10 mL/kg. Animals underwent thorough examination for the first 4 h following the dose and were subsequently assessed daily for 14 days for signs of toxicity or mortality. The therapeutic dose was defined as one‐tenth of the median lethal dose (LD_50_), calculated using Karber’s method [20]. It was categorised according to the Hodge and Sterner toxicity scale, which indicates an LD_50_ value greater than 4.0 g/kg. The LD_50_ was determined via the subsequent formula:

(1) LD50=LD100−∑a×bn.

Here, *n* = number of animals in a group, *a* = the dose difference between successive extracts, *b* = average number of deaths and LD_100_ = lethal dose.

No behavioural or physiological side effects were observed, suggesting that MR‐LEE is safe at doses up to 4000 mg/kg. Based on these findings, one‐tenth (400 mg/kg) and one‐twentieth (200 mg/kg) of the maximum tested dosage were chosen for further experimental studies.

### 2.5. Experimental Design

Rats were randomly allocated into five groups (*n* = 5 per group):

 Group I: control (normal saline, i.p.)
 Group II: GEN (100 mg/kg/day, i.p.)
 Group III: GEN + silymarin (100 mg/kg/day, p.o.)
 Group IV: GEN + MR‐LEE (200 mg/kg/day, p.o.)
 Group V: GEN + MR‐LEE (400 mg/kg/day, p.o.)

A sample size of five rats per group is sufficient to detect biochemical and histological changes in GEN‐induced nephrotoxicity, as supported by previous studies [21]. GEN 100 mg/kg/day (i.p.) reliably induced nephrotoxicity, as supported by previous rodent studies. Silymarin 100 mg/kg/day (p.o.) served as a standard nephroprotective control [22]. MR‐LEE was given orally at 200 and 400 mg/kg/day, doses chosen below one‐tenth of the LD_50_ to ensure safety [23]. GEN was administered i.p. for consistent renal injury, while MR‐LEE and silymarin were given orally to reflect therapeutic relevance. All treatments continued for 10 days, a duration sufficient to induce AKI and evaluate protective effects [22].

### 2.6. Sample Collection

An intraperitoneal injection of ketamine (50 mg/kg) and xylazine (5 mg/kg) was administered to anaesthetise the animals 24 h after the last administration [24]. Cardiac puncture was used to draw blood, which was allowed to coagulate before being centrifuged for ten minutes at 3000 rpm to separate the serum for biochemical analysis. The kidneys were carefully removed and processed for biochemical and histopathological investigation.

### 2.7. Assessment of Kidney Function Biomarkers

Serum creatinine, urea, uric acid and LDH were measured using ERBA kits (India; creatinine: MAK475A, urea: MAK471, uric acid: MAK483 and LDH: MAK 356) following the manufacturer’s instructions. The assays had detection limits of 0.1 mg/dL (creatinine), 2 mg/dL (urea), 0.2 mg/dL (uric acid) and 5 U/L (LDH), and all measurements were performed blinded [25].

### 2.8. Estimation of Oxidative Stress Parameters

Kidney tissues were homogenised in ice‐cold phosphate buffer (pH 7.4) and then centrifuged at 16,000 × *g* for 20 min at 4°C to obtain a clear supernatant for biochemical examination. Oxidative stress indicators were measured utilising known spectrophotometric methods. MDA, an indicator of oxidative lipid damage, was quantified using the thiobarbituric acid reactive substance (TBARS) method, as described by Hasan et al. [26]. Reduced GSH was evaluated by Alum et al. [27], utilising the development of a yellow‐hued compound with Ellman’s reagent. The activity of SOD was assessed by quantifying the suppression of pyrogallol autoxidation, as outlined by Alum et al. [27]. CAT activity was determined by measuring the rate of hydrogen peroxide decomposition, as described by Fouad and Ahmed [28]. All assays were conducted in triplicate, and the findings were normalised to the total protein content in the tissue homogenate.

### 2.9. Estimation of Inflammatory and Apoptotic Markers

Concentrations of TNF‐α, IL‐6 and caspase‐3 in renal homogenates were measured using rat‐specific ELISA kits (Krishgen Biosystems, Mumbai, India; catalogue numbers: TNF‐α: KRB123, IL‐6: KRB124 and caspase‐3: KRB125) following the manufacturer’s instructions. The detection limits were 5 pg/mL for TNF‐α, 4 pg/mL for IL‐6 and 0.1 ng/mL for caspase‐3 [27].

### 2.10. Histopathological Examination

Kidney tissues were fixed in 10% neutral buffered formalin, dehydrated through graded ethanol and embedded in paraffin. Sections of 4‐μm thickness were cut and stained with haematoxylin and eosin (H&E) [27]. Histopathological evaluation was performed under a light microscope (Leica DM1000, Germany) by a pathologist blinded to the treatment groups. For each sample, five randomly selected fields were analysed. Lesions were scored semiquantitatively as follows: (−) no change, (+) mild, (++) moderate and (+++) severe, based on the extent of tubular degeneration, necrosis, inflammatory infiltration and glomerular alterations.

### 2.11. Statistical Analysis

All data are expressed as mean ± SEM (*n* = 5). Normality and homogeneity of variance were checked using Shapiro–Wilk and Levene’s tests, respectively. Comparisons were performed using one‐way ANOVA followed by Dunnett’s post hoc test (SPSS Version 25). Statistical significance was indicated as ^∗∗∗^ *p* < 0.001, ^∗∗^ *p* < 0.01 and ^∗^ *p* < 0.05.

## 3. Results

### 3.1. GC‐MS Analysis of MR‐LEE

**FIGURE 1:**
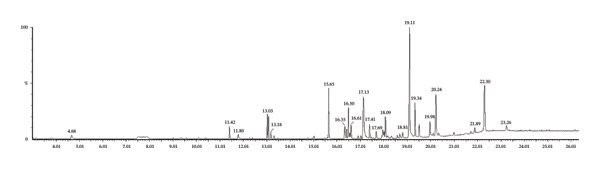
GC‐MS chromatogram of MR‐LEE. Nine bioactive metabolites were identified through GC‐MS analysis.

GC‐MS analysis of MR‐LEE revealed nine chemically diverse metabolites, predominantly consisting of fatty acid derivatives, phenolic compounds and phytosterols, as presented in Table 1 and Figure 1.

**TABLE 1:** GC‐MS identified metabolites of MR‐LEE.

No.	Identified metabolites	Molecular formula	Molecular weight (g/mol)	Conc. (%)	Retention time	Compound class	Structure
1	Methyl 10,13‐dimethyltetradecanoate	C_17_H_34_O_2_	270	6.48 25.05 23.02	11.42	Branched fatty acid ester	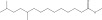
2	Methyl 9‐eicosenoate	C_21_H_40_O_2_	324	8.21	13.1	Fatty acid methyl ester	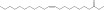
3	Linoleic acid (9,12‐octadecadienoic acid, Z, Z)	C_18_H_32_O_2_	280	12.03	13.03	Polyunsaturated fatty acid	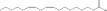
4	Hexacosyl acetate	C_28_H_56_O_2_	424	4.52	16.41	Long‐chain aliphatic ester	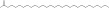
5	Coelonin	C_16_H_14_O_4_	270	7.02	17.13	Phenolic stilbenoid	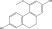
6	(E)‐3,3′‐Dimethoxy‐4,4′‐dihydroxystilbene	C_16_H_16_O_4_	272	5.03	17.41	Stilbene derivative	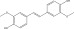
7	2,3,4,7‐Tetramethoxyfluoren‐9‐one	C_17_H_16_O_5_	300	3.79	18.09	Oxygenated polyphenol	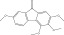
8	Ergosta‐5,24 (28)‐dien‐3β‐ol	C_30_H_50_O	426	25.05	19.98	Phytosterol	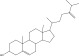
9	γ‐Sitosterol	C_29_H_50_O	414	23.02	22.3	Phytosterol	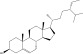

### 3.2. Acute Toxicity Test of MR‐LEE

No mortality was recorded following oral administration of MR‐LEE at doses up to 4000 mg/kg, although mild physiological toxicity was observed in one rat. Throughout the 14‐day observation period, no adverse clinical signs, including piloerection, convulsions or respiratory distress, were observed (Table 2). The high dose of 4000 mg/kg body weight was therefore considered safe and nonlethal in the experimental rats. Based on these findings, 1/10th (400 mg/kg) and 1/20th (200 mg/kg) of the maximum tested dose were selected for evaluation of the dose‐dependent therapeutic effects of MR‐LEE.

**TABLE 2:** Acute toxicity evaluation of MR‐LEE in rats.

Group	Dose (mg/kg B.W.)	Signs of toxicity/normal behaviour	Mortality/survival
MR‐LEE	1000	0/3	0/3
MR‐LEE	2000	0/3	0/3
MR‐LEE	4000	1/2	0/3

### 3.3. Body and Kidney Weight

GEN treatment markedly reduced final body weight (*p* = 0.0006) and increased kidney weight (*p* = 0.0008) compared with the control group. However, coadministration with MR‐LEE at 200 and 400 mg/kg improved these changes (*p* = 0.006 and *p* = 0.005, respectively). A similar trend of improvement was also observed in the silymarin‐treated group (*p* = 0.008, *p* = 0.007) (Table 3).

**TABLE 3:** Effect of MR‐LEE on body and kidney weight in gentamicin‐administered rats.

Group	Initial body weight (*g*)	Final body weight (*g*, Day 14)	Kidney weight (*g*, Day 14)
Control	137 ± 4.7	152 ± 3.8^∗∗^	0.91 ± 0.066^∗∗^
GEN	135 ± 4.4	127 ± 3.1^∗∗∗^	1.5 ± 0.25^∗∗∗^
GEN + MR‐LEE 200	132 ± 4.4	145 ± 2.8^∗∗^	1.15 ± 0.066^∗∗^
GEN + MR‐LEE 400	140 ± 4.4	150 ± 2.9^∗∗^	1.05 ± 0.044^∗∗^
GEN + silymarin	136 ± 2.1	154 ± 4.2^∗∗^	1.10 ± 0.036^∗∗^

*Note:* The values were reported as mean ± SEM (*n* = 5), with statistically significant differences indicated as ^∗^ *p* < 0.05, ^∗∗^ *p* < 0.01 and ^∗∗∗^ *p* < 0.001 compared with the control group.

### 3.4. Biomarker Analysis

GEN significantly increased serum creatinine, urea, uric acid, and LDH levels compared with the control group. MR‐LEE treatment reduced these alterations in a dose‐dependent manner; notably, uric acid levels declined from 5.52 ± 1.15 mg/dL in the GEN group to 4.5 ± 0.66 mg/dL and 3.90 ± 1.11 mg/dL in the MR‐LEE 200 and 400 mg/kg groups, respectively (*p* = 0.0001). These altered biomarkers showed reduced levels in a dose‐dependent manner (*p* = 0.006) upon treatments, indicating a protective effect against GEN‐induced nephrotoxicity. A similar restorative trend was observed in the silymarin‐treated group (*p* = 0.004) (Table 4).

**TABLE 4:** Effect of MR‐LEE on renal function biomarkers.

Group	Serum creatinine (mg/dL)	Urea (mg/dL)	Uric acid (mg/dL)	LDH (U/L)
Control	0.52 ± 0.04	35.8 ± 3.33	3.01 ± 0.95	3451 ± 370
GEN	1.17 ± 0.13^∗∗∗^	69.9 ± 3.44^∗∗∗^	5.52 ± 1.15^∗∗∗^	4770 ± 480^∗∗∗^
GEN + MR‐LEE 200	0.78 ± 0.06^∗∗^	55.1 ± 2.33^∗∗∗^	4.5 ± 0.66^∗∗^	4370 ± 440^∗∗^
GEN + MR‐LEE 400	0.65 ± 0.05^∗∗∗^	45.3 ± 2.66^∗∗∗^	3.90 ± 1.11^∗∗∗^	4250 ± 380^∗∗∗^
GEN + silymarin	0.61 ± 0.05^∗∗^	43.6 ± 2.8^∗∗^	3.45 ± 0.66^∗∗∗^	3870 ± 340^∗∗∗^

*Note:* The values were reported as mean ± SEM (*n* = 5), with statistically significant differences indicated as ^∗^ *p* < 0.05, ^∗∗^ *p* < 0.01 and ^∗∗∗^ *p* < 0.001 compared with the control group.

### 3.5. Inflammatory and Apoptotic Marker

GEN upregulated TNF‐α, IL‐6 and caspase‐3, reflecting pronounced inflammatory and apoptotic stress (*p* = 0.0001). MR‐LEE significantly reversed these effects in a dose‐dependent pattern, with the 400 mg/kg group approaching silymarin’s efficacy (*p* = 0.004) (Table 5).

**TABLE 5:** Effect of MR‐LEE on inflammatory and apoptotic markers.

Group	TNF‐α (pg/mg protein)	IL‐6 (pg/mg protein)	Caspase‐3 (ng/mg protein)
Control	26.2 ± 2.5	21.5 ± 2.3	16.4 ± 1.76
GEN	58.8 ± 4.6^∗∗∗^	53.1 ± 2.23^∗∗∗^	37.6 ± 2.44^∗∗∗^
GEN + MR‐LEE 200	43.5 ± 2.3^∗∗^	33.6 ± 3.22^∗∗^	29.7 ± 2.66^∗∗^
GEN + MR‐LEE 400	33.1 ± 2.3^∗∗^	29.5 ± 2.33^∗∗^	21.5 ± 1.66^∗∗^
GEN + silymarin	30.4 ± 3.0^∗∗^	23.2 ± 2.66^∗∗^	17.8 ± 1.33^∗∗^

*Note:* The values were reported as mean ± SEM (*n* = 5), with statistically significant differences indicated as ^∗^ *p* < 0.05, ^∗∗^ *p* < 0.01 and ^∗∗∗^ *p* < 0.001 compared with the control group.

### 3.6. Oxidative Stress Markers

GEN administration markedly elevated MDA levels (Figure 2(a)) and suppressed endogenous antioxidant defences, including GSH, SOD and CAT (*p* = 0.0001). Treatment with MR‐LEE restored GSH, SOD and CAT activities (Figures 2(b), 2(c) and 2(d)) and reduced MDA levels in a dose‐dependent manner (*p* = 0.008). Notably, the 400 mg/kg dose reestablished antioxidant balance comparable to that observed with silymarin (*p* = 0.005).

FIGURE 2Effect of MR‐LEE on the GEN‐induced oxidative stress. (a) Malondialdehyde (MDA). (b) Glutathione (GSH). (c) Superoxide dismutase (SOD). (d) Catalase (CAT). Gentamicin increased MDA levels and reduced antioxidant enzymes, including GSH, SOD and catalase. The 400 mg/kg dose showed effects comparable to silymarin in improving antioxidant status. The values were reported as mean ± SEM (*n* = 5), with statistically significant differences indicated as ^∗^ *p* < 0.05, ^∗∗^ *p* < 0.01 and ^∗∗∗^ *p* < 0.001 compared with the control group.

**Figure:**
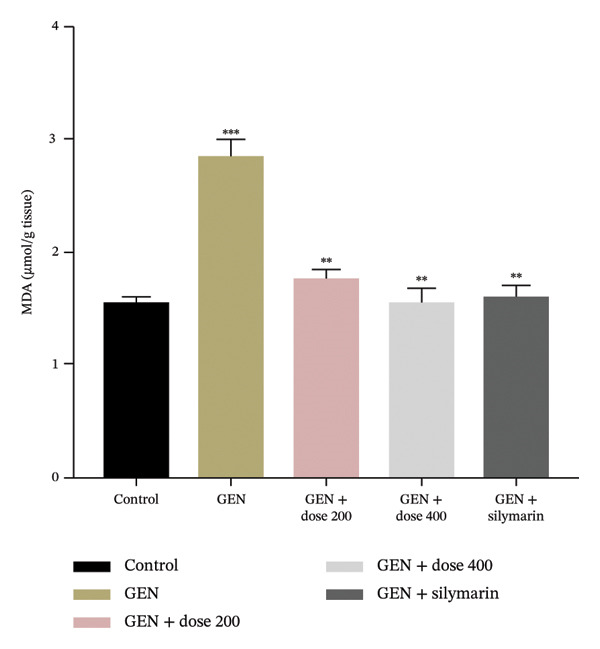
(a)

**Figure:**
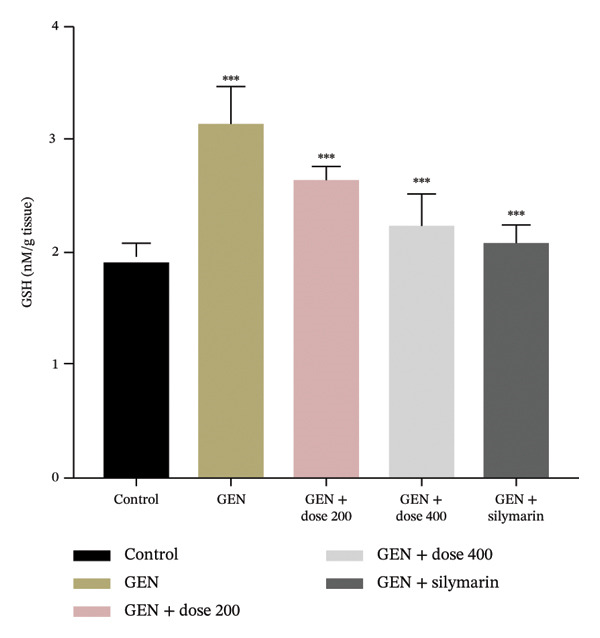
(b)

**Figure:**
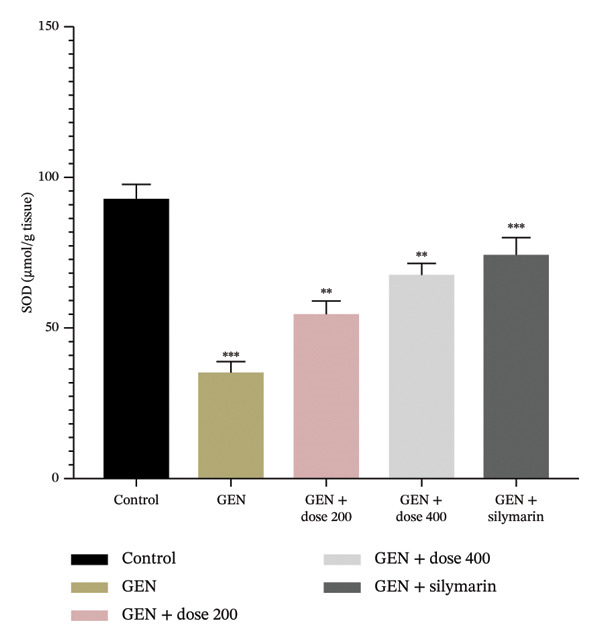
(c)

**Figure:**
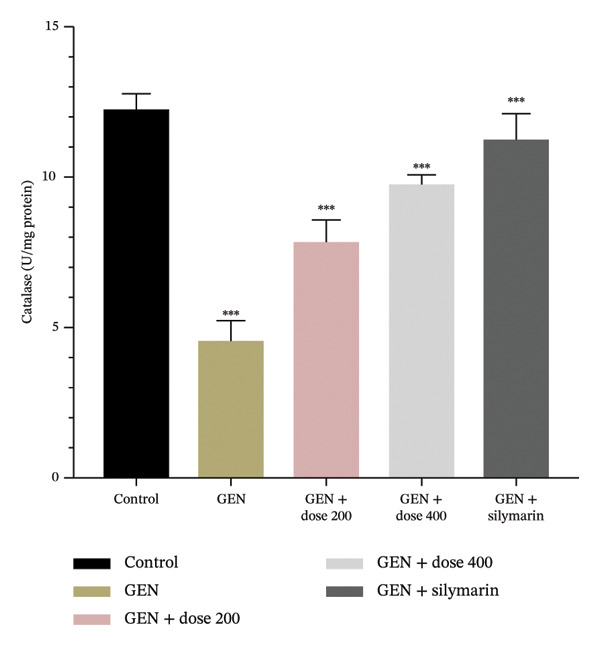
(d)

### 3.7. Histological Analysis of the Kidney

**FIGURE 3:**
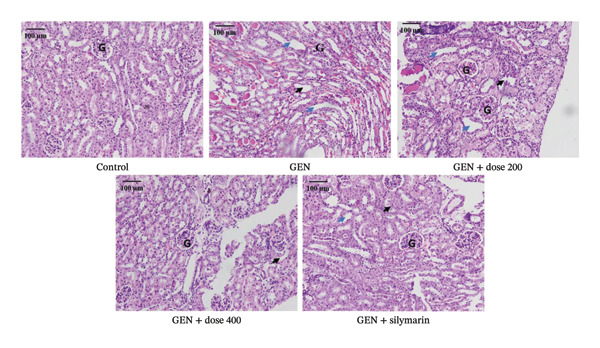
Histological analysis of the experimental groups. The gentamicin‐treated group exhibited severe renal damage with extensive tubular necrosis, tubular dilatation and inflammatory changes. MR‐LEE at 200 and 400 mg/kg further preserved renal architecture, with fewer necrotic tubules and minimal tubular dilatation. Renal damage was characterised by extensive tubular necrosis, indicated by blue arrows, and marked tubular dilatation, marked by black arrows. G: glomerulus; GEN: gentamicin.

Histological examination of rat kidney sections revealed marked structural differences among the experimental groups. The GEN‐treated group showed pronounced renal damage, characterised by extensive tubular necrosis (indicated by blue arrows), marked tubular dilatation (denoted by black arrows) and inflammatory alterations. In contrast, coadministration of GEN with MR‐LEE markedly reduced both necrotic lesions and tubular dilatation. Treatment with MR‐LEE at doses of 200 and 400 mg/kg further improved renal architecture, as evidenced by fewer necrotic tubules and minimal tubular dilatation, demonstrating substantial protection against GEN‐induced renal injury (Figure 3 and Table 6).

**TABLE 6:** Histopathological evaluation of kidney tissue.

Histopathological finding	Control	GEN	GEN + dose 200	GEN + dose 400	GEN + silymarin
Tubular epithelial necrosis	−	+++	+	–	–
Tubular dilatation	−	+++	++	+	+
Vacuolar degeneration	−	+++	++	+	+
Mononuclear infiltration	−	+++	++	++	++

*Note:* The symbols indicate lesion severity: (−) absent, (+) mild, (++) moderate and (+++) severe.

## 4. Discussion

GEN is a commonly utilised aminoglycoside antibiotic; however, its clinical use is constrained by dose‐dependent nephrotoxicity [29]. Renal damage induced by GEN is primarily linked to oxidative stress, inflammation and apoptosis in renal tubular cells [30, 31]. This study shows that MR‐LEE significantly protects against GEN‐induced renal damage in Wistar rats. The protective effects were corroborated by biochemical, inflammatory, oxidative and histopathological evidence.

GC‐MS analysis indicated that MR‐LEE contains a variety of bioactive metabolites, including fatty acid derivatives, phenolic stilbenoids, polyphenols, and phytosterols. However, the identification is library‐based unless confirmed by authentic standards. Linoleic acid and methyl eicosenoate are recognised for their ability to maintain membrane integrity and reduce lipid peroxidation, thereby protecting renal tubular cells from oxidative injury [32, 33]. Phenolic stilbenoids, including coelonin and dimethoxy‐dihydroxystilbene, demonstrate significant antioxidant and anti‐inflammatory properties and are noted for their ability to mitigate oxidative stress, inflammatory signalling and apoptotic cell death in renal tissues [34, 35]. Phytosterols, particularly γ‐sitosterol, have been shown to stabilise cellular membranes, reduce proinflammatory cytokine production and improve renal histomorphology [36–38]. Collectively, these findings may suggest that the probable constituents may contribute to MR‐LEE’s nephroprotective potential.

The acute toxicity study showed that MR‐LEE was well tolerated up to 4000 mg/kg, with no mortality and minimal physiological disturbances. This indicates a wide margin of safety and supports its suitability for therapeutic use. Based on this observation, the selected doses of 200 and 400 mg/kg were appropriate for evaluating nephroprotective activity.

GEN markedly diminished body weight and augmented kidney weight. This indicates systemic toxicity, renal inflammation and tubular oedema [29]. Comparable alterations have been extensively documented in GEN‐induced nephrotoxicity [30]. GEN significantly reduced body weight and increased kidney weight, indicating systemic toxicity with renal injury. The rise in kidney weight is mainly due to proximal tubular damage, leading to inflammatory infiltration, interstitial oedema and tubular congestion. This pathology results from GEN accumulation in the proximal tubules, which induces oxidative stress, mitochondrial dysfunction and activation of inflammatory pathways [39]. The administration of MR‐LEE increased body weight and decreased kidney weight in a dose‐dependent manner. MR‐LEE contains polyunsaturated fatty acids, phenolics and phytosterols that may protect against GEN‐induced renal injury by reducing ROS‐mediated lipid peroxidation and tubular damage [40]. These compounds may also exert antioxidant and anti‐inflammatory effects by scavenging free radicals and inhibiting NF‐κB, thereby reducing inflammation and apoptosis [41].

Renal function biomarkers unequivocally demonstrated GEN‐induced nephrotoxicity. GEN markedly elevated serum creatinine, urea and uric acid concentrations, signifying compromised glomerular filtration and tubular dysfunction. Comparable elevations have been consistently documented in experimental models of GEN‐induced renal injury, attributable to tubular epithelial damage and diminished clearance capacity [42, 43]. The significant elevation in LDH indicates compromised membrane integrity and cellular necrosis in renal tissue [44]. Treatment with MR‐LEE markedly reduced these biomarkers in a dose‐dependent manner, with higher doses exhibiting greater efficacy. This improvement may suggest preserved renal functional capacity due to protection of tubular cells from GEN‐induced oxidative stress, lipid peroxidation and inflammatory activation. GEN accumulation in proximal tubules leads to ROS generation and membrane damage, while preventing these events helps maintain tubular integrity and overall renal function [42, 43].

Inflammation is a principal factor in GEN‐induced renal damage [42]. GEN significantly elevated TNF‐α and IL‐6 levels. This indicates the activation of inflammatory pathways in renal tissue [45]. Caspase‐3 levels were elevated, signifying heightened apoptotic cell death [46]. MR‐LEE markedly diminished levels of inflammatory cytokines and caspase‐3. The effect was more pronounced at 400 mg/kg and was similar to that of silymarin. This effect may be attributed to its phytochemical constituents, including polyphenols, flavonoids, phenolic stilbenoids and phytosterols, which can suppress NF‐κB–mediated inflammatory signalling, scavenge reactive oxygen species and stabilise cellular membranes [41]. Collectively, these actions may inhibit oxidative stress–driven cytokine release and caspase‐dependent apoptotic pathways in GEN‐induced renal injury [45].

The nephroprotective profile of MR‐LEE may also be interpreted within a broader framework of acute kidney injury. Although GEN nephrotoxicity and renal ischemia/reperfusion injury differ in their initiating insults, both conditions converge on common downstream mechanisms, particularly oxidative stress, inflammatory cytokine activation and apoptosis. Previous studies showed that DHA + EPA and conjugated linoleic acid, respectively, attenuated renal ischemia/reperfusion injury by modulating antioxidant defences, inflammatory mediators and apoptotic signalling [47, 48]. The present findings, including reductions in TNF‐α, IL‐6 and caspase‐3 together with restoration of GSH, SOD and CAT, are therefore consistent with a broader body of evidence indicating that multitarget natural compounds may protect the kidney by acting on shared molecular pathways across different AKI models. Histopathological analysis validated the biochemical results. Kidneys treated with GEN exhibited pronounced tubular epithelial necrosis. Tubular dilation and vacuolar degeneration were apparent [49, 50]. Inflammatory cell infiltration was significant. These alterations are characteristic of acute tubular necrosis [45, 51]. In MR‐LEE‐treated groups, renal architecture was significantly enhanced. The severity of tubular necrosis and dilatation was diminished. Inflammatory infiltration was diminished. The dose of 400 and silymarin restored nearly normal histological characteristics, akin to those observed with silymarin.

Overall, this study’s findings demonstrate that MR‐LEE protects against GEN‐induced nephrotoxicity. Protection is mediated by antioxidant reinforcement, suppression of inflammatory cytokines, inhibition of apoptosis and preservation of renal structure and function. The synergistic action of phenolic compounds, fatty acids and phytosterols present in the extract likely contributes to these effects. Further studies are required to elucidate the precise molecular mechanisms and to support their potential translational applicability.

## 5. Limitation and Conclusion

This study provides preliminary experimental evidence that MR‐LEE protects against GEN‐induced nephrotoxicity in Wistar rats. It improved renal function markers and increased antioxidant enzyme activity. It also reduced lipid peroxidation, levels of inflammatory cytokines and caspase‐3 activity in a dose‐dependent manner. Histopathology showed better preservation of renal structure with less tubular necrosis and inflammatory infiltration. These effects may be related to antioxidant, anti‐inflammatory and antiapoptotic actions, possibly linked to its polyphenols and flavonoids. However, the study has several limitations. The sample size was small. The work was limited to a rat model. Molecular pathways were not explored. Active compounds were not isolated. There was no quantitative phytochemical standardisation. Pharmacokinetic and toxicokinetic data were not assessed. No higher animal model or human study was included. Long‐term safety and potential drug interactions were also not evaluated. Future studies should include isolation of active compounds and quantitative phytochemical analysis. Molecular mechanisms should be confirmed using genomic, proteomic and metabolomic approaches or in silico docking and pathway prediction. Pharmacokinetic and toxicological studies are also needed. Further validation in higher animal models is required. This study suggests that *M. ramentacea* has nephroprotective potential in experimental drug‐induced renal injury.

## Author Contributions

Fahmida Akter: conceptualisation, investigation, software, data curation, formal analysis, writing original draft and review and editing. Md Shafiqul Islam Sovon: investigation, software, data curation, writing original draft and review and editing. Baizid Hossain: investigation, software, writing original draft and review and editing. Md Ariful Islam: investigation, writing original draft and review and editing. Subrato Das: data curation, investigation, software, writing original draft and review and editing. Anik Biswas: formal analysis, investigation, software, writing original draft and review and editing. Md. Sohorab Uddin: investigation, data curation, writing original draft and review and editing. Md. Mahmudul Hasan: conceptualisation, investigation, data curation, writing original draft and review and editing.

## Funding

No funding was received for this research.

## Ethics Statement

The Ethical Review Board (Approval No: AERB‐FBSCU‐20250615‐(1)) approved the study, adhering to Swiss Academy of Sciences guidelines and the 2013 Animal Euthanasia Guidelines.

## Conflicts of Interest

The authors declare no conflicts of interest.

## Data Availability

The data that support the findings of this study are available on request from the corresponding author. The data are not publicly available due to privacy or ethical restrictions.
